# “Wading through the worst that humanity does to each other”: New Zealand Crown prosecutors’ experiences of working with potentially traumatic material in the criminal justice system

**DOI:** 10.3389/fpsyg.2023.1164696

**Published:** 2023-06-22

**Authors:** Rachel Kim, Nichola Tyler, Yvette Tinsley

**Affiliations:** ^1^School of Psychology, Victoria University of Wellington, Wellington, New Zealand; ^2^Faculty of Law, Victoria University of Wellington, Wellington, New Zealand

**Keywords:** vicarious trauma, potentially traumatic material, Crown prosecutors, legal professionals, lawyers, qualitative

## Abstract

**Introduction:**

Occupational exposure to trauma and its potential impacts on legal professionals working in the criminal justice system is an area that has historically been neglected and has only gained traction in recent years. Crown prosecutors, as a subset of practising criminal lawyers in New Zealand, are arguably at heightened risk of vicarious trauma (VT) due to their occupationally distinct exposure to potentially traumatic material (PTM). However, no research to date has explored the experiences of this group of working with PTM.

**Methods:**

This qualitative study aimed to explore New Zealand Crown prosecutors’ experiences of working with PTM. Nineteen Crown prosecutors from four Crown Solicitor firms across New Zealand participated in individual semi-structured interviews. The data was analysed using reflexive thematic analysis.

**Results:**

Three themes were developed that described Crown prosecutors’ experiences of work-related exposure to trauma: *trauma is everywhere, enduring effects of PTM exposure*, and *coping in the moment*. These findings add to the growing body of literature on legal professionals’ work-related wellbeing and highlights how they are an at-risk population for VT, which can be significant and enduring.

**Discussion:**

Further research is needed to understand the unique etiological pathways for both the consequences of working with PTM and effective ways to reduce this occupational risk for legal professionals working in the criminal law.

## 1. Introduction

In recent years, wellbeing has been a growing source of concern within the legal profession, as demonstrated by a plethora of popular press articles, national and international surveys, and the introduction of wellbeing toolkits (e.g., Zwisohn et al., 2018; International Bar Association, 2021; Maguire and Jarden, 2021; Owen, 2021). At the same time, research across multiple jurisdictions has demonstrated that, compared to the general population, lawyers experience high levels of psychological distress, including anxiety, depression and post-traumatic stress symptoms (Medlow et al., 2011; Leclerc et al., 2020; Weir et al., 2021). Several studies have noted psychological distress manifested as vicarious trauma (VT) in lawyers (Levin and Greisberg, 2003; Vrklevski and Franklin, 2008), described as the “transformation in the inner experience of professionals that comes about as a result of empathic engagement with clients’ trauma material” (Pearlman and Saakvitne, 1995, p. 31). Previous studies suggest that working with potentially traumatic material (PTM) can have adverse consequences such as VT, which results in altered cognitions and behaviour (McCann and Pearlman, 1990; Figley, 1995, 2002).

In this article, we focus on the experiences of lawyers working in the criminal justice system, a specialisation that consistently exposes those working within it to PTM and the trauma of others, including cases involving serious physical and sexual violence. Research with other groups of criminal justice professionals such as police officers (Parkes et al., 2019), judges (Schrever et al., 2019) and corrections officers (Campbell and Bishop, 2019) has found evidence of stress, impacts such as intrusive images, and increased risk of trauma as a result of ongoing exposure to PTM in their professional roles.

## 2. Literature review

The potential disruption to the lives of professionals as a result of experiencing VT is wide-ranging, as symptoms do not distinguish between life domains. Symptoms can present in an individual’s personal, professional and social lives (Pearlman and Saakvitne, 1995; Vrklevski and Franklin, 2008; Branson, 2019). It has been found that the development of VT is insidious, accumulating over a period of time through exposure to traumatic material (Clark and Gioro, 1998; Baird and Kracen, 2006), and is conceptualised as an inevitable occupational consequence of working with PTM (McCann and Pearlman, 1990; Pearlman and Saakvitne, 1995). The combination of potentially wide-ranging impact, accumulation over time and the prevalence and inevitability of PTM in the criminal law, all increase the importance of examining how criminal lawyers experience working with PTM and any impacts on their personal, professional and social lives.

A complex juxtaposition exists in the design of the criminal justice system; whereby through an inherently distressing process, the expectation remains for actors in the system to remain professional, emotionally detached, and impartial (James, 2008). This requirement for emotional detachment can lead to the use of denial mechanisms when it comes to acknowledging impacts on personal wellbeing (Weiss, 2009; Weir et al., 2021). On a day-to-day basis, criminal lawyers deal with cases involving serious interpersonal harm such as serious assault, rape, sexual abuse of children and murder (Murray and Royer, 2004; Vrklevski and Franklin, 2008). Occupationally, criminal lawyers are required to undertake a detailed analysis of PTM such as witness testimonies, recordings of emergency calls and photographic evidence to present their case. Additionally, criminal lawyers must also interact with traumatised individuals, ranging from those victimised by purposeful violence, to those who have perpetrated such violence, through to the historic trauma of those engaged in the process.

Criminal lawyers work within the adversarial system, which is recognised as being intense and stressful, and therefore affects and shapes criminal lawyers’ experience of their role (Murray and Royer, 2004; Seligman et al., 2005). Notwithstanding this, criminal lawyers are expected to skilfully navigate the trauma-laden criminal system and remain unscathed. This expectation rests on the failed premise that lawyers are *“cold”, “heartless”* and *“robotic”* beings that exert rational and unhindered judgement (Daicoff, 2004; Vrklevski and Franklin, 2008). However, there is growing evidence to suggest that criminal lawyers experience and are impacted by traumatic material and emotion as part of their role (Murray and Royer, 2004; Vrklevski and Franklin, 2008).

Limited studies have explored the impact of working with PTM among lawyers. A recent scoping review conducted by Léonard et al. (2020) identified only nine original, quantitative investigations of work-related PTSD among lawyers. Likewise, only two qualitative studies were identified that investigated the experiences of criminal lawyers working with PTM (e.g., Gomme and Hall, 1995; Weir et al., 2021). However, the small number of studies that do exist suggest that criminal lawyers are occupationally at risk of being affected as a result of working with PTM and report negative symptoms (e.g., Levin and Greisberg, 2003; Vrklevski and Franklin, 2008; Maguire and Byrne, 2017; Weir et al., 2021). For example, Levin and Greisberg (2003) found that criminal lawyers in the United States had higher levels of burnout than social workers or mental health professionals, in part because of the combination of high caseloads and low supervisor input on the effects of working with PTM. Similarly, Vrklevski and Franklin (2008) found that Australian criminal lawyers suffered more VT, stress and other impacts of working with PTM than those working in civil law specialisations.

Prior to this study, no New Zealand research focusing specifically on criminal lawyers has been reported. In addition, there has been no research that looks at the different experiences of legal professionals depending on the role they play within the criminal justice system. In New Zealand, criminal lawyers tend to work exclusively for the defence or prosecution at any one time. Therefore, the experiences of defence counsel and prosecutors of working with PTM and its effects may differ. Further, among prosecutors, the experience of Crown prosecutors, who work with more serious cases, may also differ from that of Police prosecutors, who hand over more serious cases to the Crown. Through their responsibility to prosecute more serious criminal offending on behalf of the Crown, Crown prosecutors are arguably expected to deal with cases that are inherently more distressing and traumatic compared to other legal professionals, placing them at heightened risk of the adverse consequences of repeated exposure to PTM.

The significant impacts of PTM exposure on prosecutors working in the criminal justice system and the responsibilities of employers (including the State) to reduce employees’ risk of psychiatric injury has recently been demonstrated in the High Court of Australia case *Kozarov v State of Victoria* (Zagi, 2022), where the State of Victoria was found to have breached its duty of care to a prosecutor in its Specialist Sexual Offences Unit. However, no studies to date have sought to investigate the occupational risks of New Zealand Crown prosecutors in working with PTM. Thus, this study sought to qualitatively explore the lived experiences of Crown prosecutors in their work with PTM and its consequences, in the unique context of the criminal justice system. A qualitative approach was adopted to enable an in-depth and rich understanding of Crown prosecutors’ individual experiences of working with PTM. Increased knowledge about the experiences of those working within the system is a vital step toward the introduction of measures by employers and professional bodies to reduce levels of VT and other forms of psychological distress. Through in-depth interviews with Crown prosecutors who work predominantly in criminal law, our study aims to improve understanding of the exposure to, and impact of, PTM for those working in the criminal justice system, by asking the question: what are Crown prosecutors’ experiences of working with PTM?

## 3. Materials and methods

### 3.1. Design

A phenomenologically oriented qualitative design was adopted for the current study as we wanted to understand, prioritise, and give voice to Crown prosecutors’ personal experiences of working with PTM from their own perspective (Braun and Clarke, 2006, 2013). A semi-structured interview schedule including broad open-ended questions was used to encouraged participants to discuss their experiences in their own words. Analysis was informed by a critical realist epistemology and conducted using an inductive and semantic approach. This means that themes were developed directly from the data and explicitly reflect prosecutors’ interpretation of their reality in their professional role, within the broader social context of the criminal justice system; as opposed to being interpreted through a particular theoretical lens (Braun and Clarke, 2006).

### 3.2. Participants

Participants were 19 practising Crown prosecutors (7 male, 12 female) working in the criminal jurisdiction in New Zealand. Participants were recruited from across four Crown Solicitor firms. Participants’ ages ranged from 25 to 55 years (*M* = 35.11, *SD* = 9.93) and the majority identified as Pākehā/European (84.2%). Approximately half (47.4%) of participants were Junior Crown prosecutors, and the length of time spent in their current role ranged from 4 months to 27 years (*M* = 7.5 years). See Table 1 for an overview of participant demographics.

**TABLE 1:** Participant demographics.

Demographics	Category	*N*	%
Gender	Male	7	36.8
	Female	12	63.2
Ethnicity	Pākehā/European	16	84.2
	Māori	2	10.5
	Asian	1	5.3
Classification^1^	Junior	9	47.4
	Intermediate	4	21.1
	Senior	6	31.6
Provincial/Urban	Provincial	12	63.2
	Urban	7	36.8

^1^The full classification criterion for a junior, intermediate and principal prosecutor can be found in the Crown Solicitors: Terms of Office (2017).

### 3.3. Procedure and materials

This study was conducted as part of a larger project examining New Zealand Crown prosecutors’ experiences of working with PTM and emotion in the criminal justice system. Ethical approval was obtained from the Victoria University of Wellington Human Ethics Committee (Reference: 28052). Crown solicitors’ firms were contacted via email and those who expressed an interest in sharing the research with their staff were then sent an email invitation and information sheet to disseminate to all staff practising as Crown prosecutors in the firm. Prosecutors who were interested in participating in the research contacted the research team directly to arrange a date and time for an interview. All participants provided written consent prior to commencing their interviews.

Interviews were conducted by the first author via audio-visual link (Zoom). Interviews were initially planned to be conducted face-to-face or via telephone; however, this was changed to audio-visual link due to the COVID-19 pandemic. Although the interviews could not be conducted face-to-face, the use of audio-visual links for interviews is not unprecedented with this participant group (see Britton, 2018) and has several advantages including increased privacy and anonymity for small participant groups, and flexibility in terms of interview times and locations (Sturges and Hanrahan, 2004; Holt, 2010).

A semi-structured interview schedule was used to guide the interview process. The interview schedule comprised five sections: (1) demographic and background information, (2) participants’ role as a prosecutor, (3) working with potentially traumatic material, (4) managing emotion in the workplace, and (5) workplace support and wellbeing. Interviews ranged from 42 minutes to 2.5 hours in length (*M* = 57 minutes). At the end of the interview, all participants were sent a debriefing sheet via email which included information about free support services. During the interview, participants were given the option to stop the interview and take a break or withdraw from the study if they reported or were observed to be visibly upset, and details of free support services/helplines provided verbally.

All interviews were recorded using the meeting recording function in Zoom and transcribed verbatim by the first author. To maintain confidentiality, participants were allocated a pseudonym and any potentially identifying information (e.g., places, names, specific experiences) were redacted. Participants were also offered the opportunity to review their transcripts and highlight any information that they wished to be redacted or excluded from the research.

In addition to ensuring participants’ safety and wellbeing, there was similarly a need to protect researcher wellbeing throughout the research process, due to the nature of the work of Crown prosecutors and the scope of the current study. Debriefing and supervision with a senior member of the research team was available following each individual interview, to provide a space to discuss any issues that arose, points of distress, and connection with appropriate support services if required. Regular reflective supervision was also provided throughout transcription and analysis, where prolonged immersion with the data is required.

### 3.4. Data analysis

Interview transcripts were analysed using Reflexive Thematic Analysis (RTA) following Braun and Clarke’s (2006, 2013) six step process. An inductive and semantic approach to analysis was used. This means that analysis was not guided by a theoretical framework and that themes were developed to reflect the explicit content of the data and participants’ experiences as described in their own words. The six steps of RTA, as outlined by Braun and Clarke (2006, 2013), were completed as follows:

1) *Data familiarisation:* This involved the researcher immersing themself in the data. This was achieved through two means: the manual transcription of the interviews and the reading and re-reading of each participant’s transcript. Notes of initial thoughts and observations were also made at this stage.
2) *Generating initial codes:* Initial codes were developed through identifying segments of text relevant to the research question within the transcripts and then ‘tagging these’ with a summary description or phrase that reflected the central meaning of what was being said (Braun and Clarke, 2006, 2013). Coding was an iterative process that involved continued refinement. Once finalised, all codes and relevant data extracts from across the dataset were collated in a table and further refined to ensure these accurately captured and reflected participants’ intended meaning.
3) *Searching for themes:* Codes and their exemplary quotes were printed, reviewed, and then physically arranged into provisional groupings (preliminary themes) based on their shared meaning or connectedness.
4) *Reviewing themes:* The cohesiveness and coherence of the preliminary themes were reviewed against data extracts (i.e., quotes from the interview transcripts) and each other to ensure each theme accurately reflected participants’ accounts and were distinct one from another (Braun and Clarke, 2006, 2013). A thematic map was also produced at this stage to examine the relationships between the themes and their subthemes.
5) *Defining and naming themes:* Due to the cyclical nature of RTA, the researchers moved back and forth between stages four and five, to identify the “essence” of each theme and organise the data extracts to form a coherent and consistent narrative of the experiences of Crown prosecutors (Braun and Clarke, 2006, 2013). This was an iterative process of rearranging subthemes and themes to ensure the best fit, and then naming these based on their core defining features. Again, this was an iterative process that required going back to the original meaning intended by participants when describing their experiences to ensure theme names captured and expressed what was reported in the data.
6) *Producing the report:* A written narrative was produced that described each of the themes and subthemes developed through the analysis, exemplified by supporting quotes. Further refinements were made to the results during the report writing process to ensure these accurately communicated participants’ experiences.

### 3.5. Reflexivity

The research team was comprised of three female researchers, with academic backgrounds in law and psychology. The first and third author have both worked as legal practitioners in the criminal law in the United Kingdom and New Zealand, and the second author has a background working in forensic settings. Interviews and data analysis were led by the first author, who kept a reflective journal throughout the research process. This journal was used to reflect on initial thoughts and interpretations of the data, and the researcher’s position in the research during analysis. Regular supervision meetings were held throughout the research process to maintain the wellbeing of researchers. Supervision meetings were also used to reflect on how our characteristics and previous experiences may influence interpretation of the data, to triangulate thinking and consider alternative perspectives, and to ensure participants’ voices were being centred. Through this process, refinements were made to code and theme names and descriptions, and the interpretation of the findings. For example, given our own experiences of working in the criminal justice system, both as academics and practitioners, it was important to exercise care during analysis so as not to inadvertently dismiss an experience of a participant by labelling it as “typical” of working in that context.

## 4. Results

Three themes were developed from the data that described Crown prosecutors’ experiences of working with PTM in the criminal justice system. These were named *trauma is everywhere, enduring effects of PTM exposure*, and *coping in the moment.* See Table 2 for overview of the themes and subthemes.

**TABLE 2:** Themes and subthemes.

Theme	Subtheme
1. Trauma is everywhere	1.1. The multi-modal and multi-sensory nature of PTM exposure
	1.2. Hierarchies of trauma, offending, and responsibility
	1.3. The emotionality of working with complaints
2. Enduring effects of PTM exposure	2.1. Exposure to the “real” New Zealand
	2.2. Sense of responsibility to complainants
	2.3. Personal connections to files are triggering
	2.4. Unable to leave work behind
	2.5. Impact on personal relationships
3. Coping in the moment	3.1. Passive acceptance
	3.2. Survival through desensitisation
	3.3. Maintaining professionalism

### 4.1. Trauma is everywhere

This theme outlines the nature and extent of Crown prosecutors’ exposure to PTM on a day-to-day basis and how this permeates every aspect of their role; resulting in continuous and cumulative exposure to PTM and a feeling of being surrounded by trauma. Three subthemes were developed that described the different types of PTM Crown prosecutors were exposed to and their experiences of interacting with this PTM: *the multi-modal and multi-sensory nature of PTM exposure; hierarchies of trauma, offending, and responsibility*; and *the emotionality of working with complaints.*

#### 4.1.1. The multi-modal and multi-sensory nature of PTM exposure

Crown prosecutors detailed their occupational exposure to PTM and noted their observations that *trauma is everywhere* in their role. Material containing distressing or traumatic content that evidenced criminal offending was reported as the most common PTM experienced and was described as being both *multi-modal*, in that it was presented to prosecutors through various mediums, and *multi-sensory* in nature, due to the distinct sensory interactions Crown prosecutors had with this material:

“…*post-mortem homicide photos, listening to 111 phone calls of people requiring help because some crime is currently being committed, photographs of injuries, doctor’s descriptions of injuries, child pornography, victim impact statements. Just to name a few.”* – Jamie

In addition to file based PTM, Crown prosecutors also described being exposed to the trauma of others through attendance at post-mortems and crime scenes as well as interactions with complainants and their families.

“…*we’re exposed to trauma through the narratives of people’s traumatic experiences, both in terms of recorded media, written statements and by our personal interaction with them as well as the evidence that they give.”* – Sam

This in person exposure was described as being unique to the Crown prosecutor role and was identified as more traumatic than retrospective file or photographic information:

*“Probably the only difference is that for the Crown, there are some times where you have to physically attend the post-mortem or you might actually have to go to the crime scene and*… *the deceased may still be at the crime scene which probably would be unusual for a defence lawyer to be present at those usually. And I think the physically seeing these things*…*it seems to, I think that’s more traumatic than seeing photos.”* – Morgan

It was apparent that Crown prosecutors normalised this exposure to trauma in their professional lives, with PTM described as *“material that ends up on our desk in one form or another or in a courtroom live”*, highlighting the inevitability of PTM exposure in their role.

#### 4.1.2. Hierarchy of trauma, offending, and responsibility

Crown prosecutors described three hierarchies that captured the nature and severity of the types of offending and traumatic content that they work with. Due to the nature of cases delegated to Crown Solicitors, Crown prosecutors described predominantly working with serious drug offences and offences involving individuals who have experienced sexual or interpersonal harm. These offence categories were viewed by Crown prosecutors as “high” level and more serious than those other groups of prosecutors worked with within the New Zealand criminal justice system. Within these “high” level offences, prosecutors reported that as they gained experience and seniority in their role, they were allocated increasingly serious offences (e.g., homicide and sexual violence) which came with an increased level of responsibility and trauma exposure.

“… *you are then doing more high-level kind of work. More complex files or files involving a higher degree of seriousness in terms of the alleged offending*… *So the nature of the work changes, shifts as you become more senior and you have more responsibility*.” – Sam

Cases involving complainants harmed through sexual or violent offending were described as ranking higher on the trauma, responsibility, and offending hierarchies. However, sexual violence was overwhelmingly regarded as the most traumatic offence type to work with. Evidence associated with sexual violence cases was described as having a *“unique edge”* and elicited a particularly strong emotional response, with participants describing this as *“disgusting”, “gross”* and *“graphic”*, and particularly disturbing to work with:

*“It’s really challenging. Especially in the sexual violence space because it gets really disgusting, you know? You’re hearing details of things that you don’t want to think about.”* – Mackenzie

Sexual violence cases were also described as involving higher levels of trauma exposure and emotional impact both because of the evidentiary material that Crown prosecutors were exposed to in these cases but also because of the trauma experienced by complainants and their families:

“…*listening, being with victims who have been sexually abused I find that traumatic and seeing the effect upon them. So not so much what occurred, I can process what occurred, I find it traumatic seeing the impact of that upon people’s lives.”* – Billie

#### 4.1.3. The emotionality of working with complainants

The Victims’ Rights Act 2002 and the Solicitor-General’s Prosecution Guidelines 2013 require Crown prosecutors to meet with complainants during the trial process in violent offending cases. For more senior long-serving Crown prosecutors, who lead prosecutions on the most serious cases, the introduction of these requirements resulted in increased contact with complainants than what was expected previously. Many participants (both junior and senior prosecutors) described direct interpersonal contact with complainants as the most challenging aspect of their role as it created a personal connection with an individual who had previously been viewed through the more distanced lens of a case file. Whilst paper-based evidence could be reappraised as factual and treated in a more clinical manner, witnessing the lived trauma of an emotional complainant was described as confronting and much more difficult to distance oneself from:

*“I mean a lot of the time like I said my files are on paper and it’s quite factual and evidence based, so sometimes it’s not even emotional because it’s just the way it’s worded is very factual and the way you’re looking at it is very factual and it’s all on paper and it’s just pictures. It’s not in front of you and*… *you can separate the pain from the evidence. So I find that okay*… *and then sometimes it’s harder when you meet the people and then obviously you can see the pain on their face*.” – Ash

Preparing for trial was described as particularly stressful and involving increased exposure to PTM, as the process requires in-depth analysis of file based PTM as well as meeting with complainants and their families. The trial preparation process was also reported to heighten Crown prosecutors’ awareness of the adverse consequences of the offending for complainants which would leave them feeling emotionally affected.

“… *it’s not emotional in the first instance but it can become emotional. You can sort of have emotions well up inside of you later on down the track when you start to*… *get all the information and if it’s going to trial*… *you have to deal with it more in depth. Then you’re looking at pictures that they’ve been badly beaten*…” – Danny

Although the Crown prosecutors acknowledged the necessity of trials for the functioning of the criminal justice system, they also described how the trial process inadvertently contributed to the re-traumatisation of complainants and the *sense of responsibility* they felt for putting complainants through this.

*“You know, you can see them clench their fists and dig their nails into their palm to the point that it’s just about to bleed or is. You can see them crying. You can see the effect on them*… *and you know you have to ask the questions because it’s part of that process. So in a way, you’re part of inflicting the trauma as well*.” – Sam

Coming face-to-face with others trauma through interactions with complainants and the trial process often led to emotional transference that invoked both a *sense of responsibility* and emotional impact on Crown prosecutors.

### 4.2. Enduring effects of PTM exposure

This theme describes the different ways exposure to PTM impacted Crown prosecutors’ emotional and mental wellbeing and comprises five sub-themes: *exposure to the “real” New Zealand*, a *sense of responsibility to complainants, personal connections to cases are triggering, feeling unable to leave work behind, impact on personal relationships.*

#### 4.2.1. Exposure to the “real” New Zealand

This subtheme describes Crown prosecutors’ journey in their understanding of the existence and frequency of serious crime in New Zealand and the impact this had on their view of the world and themselves. Participants typically described their upbringing as sheltered and having lived in their “*own middle-class bubble about what is going on in the world*”. As a result, participants found it confronting being exposed to “the underbelly” or the “real” New Zealand:

*“Before I made it, people had told me be prepared of the chaos of the District Court, procedurally. People won’t show up, people will be late. But I don’t think I had subject matter in much detail. So I grew up in a wonderful sheltered lovely home (laughs) and very quickly I realised I had experience the tip of the iceberg of life experience*.” – Alex

Systemic issues and patterns of crime such as the over-representation of Māori in the criminal justice system, intergenerational trauma and criminality, and social disadvantage were particularly poignant realisations for participants:

*“That sort of changes your view on the world, and knowing there’s so many people who come from such disadvantaged and dysfunctional backgrounds*… *You can see cycles within families and that’s really sad*.” – Jessie

Through exposure to such shocking realities, Crown prosecutors developed a self-realisation of their own privilege, reporting *“feeling lucky”* in their own upbringing and life experiences:

*“Being exposed to the participants in the criminal justice system who lead quite unfortunate lives, I suppose it reinforces how privileged I have been and am.”* – Jamie

Thus, while Crown prosecutors reported their confrontation with the “real” New Zealand as unexpected, shocking and stressful, it also allowed participants to recognise their own privilege and feel fortunate for their own life experiences.

#### 4.2.2. Sense of responsibility to complainants

Meetings with complainants were often described as leading to the formation of a professional relationship as well as an increased awareness of the impacts of the offending, reinforcing the gravity of “losing” a trial for the complainant. As a result, Crown prosecutors discussed feeling an overwhelming *sense of responsibility* toward complainants and their families to get a desirable outcome:

*“Unfortunately, the more you empathise with and the more you get to know your victims and their families, the more pressure there is, you know, the more pressure you actually feel to want to get a result for them*.” – Mackenzie

This heightened sense of responsibility meant that when Crown prosecutors did not get a guilty verdict, they personally felt distress, disappointment and guilt:

*“I would cry and cry and cry and think it was my fault and then you’d go talk to another prosecutor and they would say “well it’s not about the verdict. The verdict’s irrelevant. It’s not about the verdict. Your job is to present the case*… *Juries are fickle and juries are strange beasts and they come up with all kinds of crazy decisions*… *So you can’t take it personally”. But how can you not? You’ve invested hundreds of hours into this case, met the person, they’re you know, completely given their life’s story and they’ve spoken to you about the most traumatic thing that’s happened to them and then you’re meant to say, “oh well I did my best”, and walk away and move onto the next one*.” – Jessie

The sense of personal responsibility led to a negative cycle of self-criticism for several Crown prosecutors, who described feeling like an *“idiot”* or a *“loser”* when a not guilty verdict was returned. In more extreme cases, this duty to complainants appeared to subsume the basic human need to self-protect, with prosecutors explicitly acknowledging working at their own detriment to shield complainants through the criminal justice process:

*“I think you have to tip the scale in favour of them because they didn’t sign up to be in this system. So you just really have to do the best job for them and then just cope with it later. You know, it’s not their fault that you feel, how you feel is not their fault. Whereas I can control how they feel so yeah. I think you just have to put yourself to one side, sort of block it off and try not to think about it too much*.” – Mackenzie

Whilst this *sense of responsibility to complainants* was often viewed as a motivational factor to do well in the role, it also increased the pressure to get a favourable outcome and negatively impacted the wellbeing and personal functioning of Crown prosecutors.

#### 4.2.3. Personal connections to files are triggering

Crown prosecutors described how having to work on cases that reflected aspects of their own lives as particularly challenging. These cases could also trigger personal trauma particularly when there were similarities in facts or details that mirrored their own lives and experiences (e.g., names, type of offending, child complainants of similar ages to their own children):

*“It was way too familiar. And it was just a real, to be able to identify so closely with*… *because you’re going through and you’re reading the report and the photos of the scene, it all came flooding back.”* – Jordan

Cases which reflected personal issues in Crown prosecutors’ own lives were described as more traumatic to work with than cases that did not contain these connections but would typically be viewed as having inherently higher trauma content; suggesting that material with “gruesome” or objectively high trauma content, in and of itself, is not the only pathway for Crown prosecutors to experience (or reexperience) trauma in their role:

*“You don’t forget about those files. The names of every single one of them*… *With the murders though, I’ve reviewed several murder files now, I don’t have that same reaction. Because even though it’s traumatic, somebody’s died and they often die in gruesome ways, that doesn’t trigger me nearly as much.”* – Jordan

#### 4.2.4. Unable to leave work behind

Prosecutors expressed how the content and nature of their role permeated all aspects of their life reflected in an inability to “switch-off” after and outside of work, a pre-occupation with material they had been exposed to, intrusive thoughts, and an inability to sleep:

*“Certainly, I know that I used to have trouble sleeping and find myself really preoccupied with things that I read and heard about and they would recur at all kinds of times when you’d just be trying to engage in normal family or social life*… *I do think it really contributed to my depression.”* – Mackenzie

In addition, participants reported having an increased sense of cynicism and suspicion toward other people as a result of the material they were exposed to in their role:

*“I suppose like when I started the expectation was that crime was committed by baddies and over time you realise that crime is committed by everyone everywhere, and you can’t ever you sort of lose trust in people and everyday circumstances.”* – Jordan

This increased cynicism and suspicion sometimes manifested as a heightened sense of paranoia and fear for their own and loved one’s personal safety. Although the illogicality of the paranoia was acknowledged, Crown prosecutors described how it could not easily be overlooked:

*“I think another way that it’s affected me which seems ridiculous to my logical mind right? We own a house, it’s a nice enough area but my [partner] often works night shifts so sometimes I’m home alone at night. And I’ve never been scared of being home alone at night. But*… *often I would say, a handful of times in the last year*… *when I’ve woken up in the night, I’ve thought is there someone in the house? Could someone come in the house? There could be someone going to do a burglary. There could be someone coming in to look for [someone] to rape*… *It’s (pause) a fear or an anxiety that I never had before. And I’m like it’s not going to happen, you shouldn’t feel like this, calm down. But eventually I always get up and turn a light on (laughs).”* – Alex

This meant local areas and normal recreational tasks became potentially triggering for Crown prosecutors, due to their heightened awareness of crime within the local community. Thus, leading a “normal life” was described as no longer possible:

*“So like before you know, I used to drive past camp grounds at the beach*… *and think, oh great, these people are having a nice holiday. And now I drive past a campground and I think, there’s definitely going to be at least ten kids getting molested in there. It’s impossible to not look at ordinary situations and now you know that in fact, there’s a lot of offending in all sorts of different contexts going on*.” – Mackenzie

Whilst some Crown prosecutors expressed a level of helplessness toward these impacts (e.g., its “typical of the job”), others reported taking a more active approach, attempting to adjust their skewed world-view “*to not be so pessimistic about everything*”.

#### 4.2.5. Impact on personal relationships

The role of a Crown prosecutor was often described as *“all-consuming”*, which was compounded by the personal impacts of working with PTM as described in the previous subtheme. Participants reported that the exposure to PTM in their job as well as the all-consuming nature of their role had a negative impact on their relationships with loved ones (i.e., significant others and family members). Crown prosecutors reported having limited emotional capacity for others due to the overwhelming stressors in their professional lives, with continuous interaction with PTM reported as the most prominent stressor. This impacted on the quality of personal relationships and, at times, led to the breakdown of relationships:

*“It’s an insane job*… *I’m just not emotionally available to anyone and when you are in a stressful kind of job, you do shut out other aspects of your life. So yeah. I’ve definitely had relationships that haven’t worked because I just don’t have any emotional bandwidth to give the other person*.” – Bailey

Participants described the trial process as a significant pressure point in their roles rendering them incapable of carrying out basic household responsibilities such as cleaning or cooking due to the workload and needing to rely on loved ones to take these on for them. While emotional unavailability could stem from both general work stressors and PTM exposure, physical unavailability (i.e., difficulty with physical intimacy) was particularly associated with high caseloads of sexual violence offending. When working with sexual violence cases, Crown prosecutors described that being physically intimate was impossible due to the rigour of analysis of PTM for trial and fear of triggering visualisations or thoughts:

*“If I’m in trial or the lead up to a trial, my job is to vividly (laughs) paint a picture of sexual violence. My head is busy trying to recreate a picture of sexual violence. Dealing with inconsistencies in evidence*… *And it’s stewing over that in my subconscious and there is no way, well, to date, no way I’ve found to be physically intimate with my partner.”* – Alex

An additional impact on Crown prosecutors’ personal lives was the fear of starting a family. This pertained to fears of parental inadequacy due to the stress and demanding nature of the role, which left little mental and physical capacity to look after a child:

“*I don’t know if I could be present as a parent at the end of a work day when I’m in trial or days when it’s that stressful*.” – Alex

It was evident that the impact of Crown prosecutors’ occupational exposure to PTM was far reaching and impacted both themselves and inadvertently, their loved ones, and their family decision making.

### 4.3. Coping in the moment

Crown prosecutors discussed using a variety of coping strategies when working with PTM. These strategies often represented short-term measures to protect wellbeing and maintain professional standards including *passive acceptance, survival through desensitisation* and *maintaining professionalism.*

#### 4.3.1. Passive acceptance

Crown prosecutors routinely acknowledged their role involved exposure to PTM and the inevitability of becoming affected to some degree due to their continuous occupational exposure. However, they also tended to downplay their negative experiences of working with PTM, relabelling these as something that was “part and parcel of the job”:

“… *unfortunately, everyone seems to accept that’s just the process you’re going to have to go through and that’s the journey of a Crown prosecutor.”* – Blake

Passively accepting the negative impacts of the job appeared to be one way in which Crown prosecutors attempted to normalise and make sense of the hardships encountered in their role by framing these as “typical” of the job. This normalisation occurred, in part, through shared experiences with their colleagues. Crown prosecutors described finding comfort among their colleagues in having experienced similar negative experiences, as it normalised their own feelings of confusion and distress. However, this also contributed to the creation of a safe and supportive work environment (a protective factor against VT):

*“Every time I’m having a bad day at work*… *like haven’t had good sleep or stressed*…*and [the Crown Solicitor] says you know, if you need to talk about stuff, I can 100% guarantee you that I’ve been through it or worse you know? So it’s really just comforting to know other people are going through or have gone through the same thing. And are there to look after you*.” – Danny

#### 4.3.2. Survival through desensitisation

Crown prosecutors often spoke of the “gold standard” of desensitisation and their desire to reach this to survive long-term in their role. Partners in the firm were often perceived as *“immune”* or *“hardened*” to the point that PTM no longer affected them and failed to display any vulnerability as prosecutors. Participants detailed the difficulties of working with PTM and spoke of wanting to reach a point where they no longer felt the negative impacts:

*“I’m hopeful that in ten more years, I’ll be a little less affected like my bosses are (laughs).”* – Mackenzie

The need to become desensitise was reinforced when partners failed to display empathy and support for staff members who were struggling with the content of PTM and instead, minimised their experiences. Perhaps whilst not intentional, this displayed to participants that having an emotional reaction to PTM was viewed as “weak” and undesirable:

*“I’ve heard horror stories about partners not really being able to relate to people who are genuinely distressed*… *at the end of a trial and all the constructive feedback being able to be something along the lines of like “oh don’t get too worked up about it” or something like that*…” – Blake

However, in contrast to the above, some Crown prosecutors refuted the idea of a textbook “robot prosecutor” that is void of emotion, expressing their belief that emotional intelligence was integral for a Crown prosecutor. This was due to the human interactions and relatability required in the role:

*“I don’t think a prosecutor can be a robot. Because our job is to relate to complainants and witnesses in a way that acknowledges the experience they’ve had, which is often tragic, and we need to relate to the jury as a person.”* – Alex

Desensitisation was viewed by these individuals as a potential hindrance to a prosecutor’s ability to be an effective advocate for complainants and victims:

*“I think if you get blasé, or you get so to the point where you’ve heard the story so many times and it doesn’t affect you, I think you lose a little bit of your ability to become a good prosecutor. You certainly lose your ability to talk to and look after victims I believe.”* – Billie

#### 4.3.3. Maintaining professionalism

Crown prosecutors are required to maintain professional boundaries in their interpersonal interactions with complainants and victims. Participants reflected that in order to uphold their duty to be a fair and impartial prosecutor, they had to maintain a level of emotional distance. However, in practice, participants described grappling with professional boundaries due to the rawness of the human interactions involved and the *emotional transference* and *responsibility* experienced toward complainants. Strictly enforcing distance between themselves and distressed individuals was described as a necessary but unnatural strategy to maintain these boundaries:

*“You can’t give them the impression that you’re their lawyer. You cannot at all talk to them about the evidence. So you go in there and you effectively say nothing to them. And you know that person’s about to go into court and give evidence about the hardest thing that’s ever happened to them and you have to sort of switch off and say, just let me know if you need a break and we can stop at any point and you know, if you have any questions ask the officer in charge because I can’t talk to you (laughs)*… *I find that incredibly difficult.”* – Jessie

Achieving the “appropriate” level of boundary setting was described as a constant and challenging process of trial and error:

*“Look it’s a constant struggle. Sometimes I feel like I’ve done it okay, sometimes I feel like I’ve messed it up entirely*… *you want them to feel like you are listening to them and you’re there for them and you know them, but you also have to maintain that professional, objective role of a prosecutor. So you don’t want to get too emotional about it. But yeah, it’s honestly probably the hardest, one of the hardest things about the job, is striking that balance correctly.”* – Bailey

Maintaining professionalism through difficulties encountered in the role, particularly in terms of complainant/victim interactions, protected Crown prosecutors from being drawn into an emotional response. However, this required a large amount of emotional labour due to the active conflict with “switching off” natural human responses (e.g., empathy, sadness) when interacting with distressed individuals.

## 5. Discussion

This study aimed to investigate the experiences of New Zealand Crown prosecutors in working with PTM. Participants described feeling *trauma is everywhere*, reporting particularly marked impacts for those working with prolonged exposure to offences involving interpersonal violence. While prosecutors expressed difficulties in managing the relationship between empathy for complainants and the law’s requirements of emotional detachment, it is apparent that they must navigate this alone through a process of trial and error. Many prosecutors reported that their work had negatively affected their relationships and their view of the world and human behaviour, and the majority passively accepted the inevitability that they would need to continue to deal with trauma and its impact on their lives and their world view. Most participants reported desensitisation as either a result of their work or for some, a goal to be strived for.

Consistent with previous studies exploring the experiences of criminal lawyers, Crown prosecutors acknowledged persistent and prolonged exposure to trauma through their work in the criminal justice system (e.g., Maguire and Byrne, 2017; Weir et al., 2021; Birze et al., 2022a). Unique to the role of a Crown prosecutor was the heightened exposure to serious physical and sexual violence. Working on cases involving sexual offending was identified as most traumatic; mirroring the findings of Weir et al. (2021), where Australian lawyers described sexual abuse, particularly involving children, as a distinct stressor in their work. Further, research police officers has found that working with sexual trauma heightens the risk of VT and compromises psychological wellbeing (e.g., Perez et al., 2010; Powell et al., 2014, 2015).

Crown prosecutors identified meetings with traumatised complainants and victims as highly traumatic, due to the emotional transference that occurred from such individuals to prosecutors. Continued contact with complainant/victims was reported to foster an environment where Crown prosecutors were expected to build rapport and empathic engagement that mirrored a therapeutic relationship, which has been identified as a gateway to developing VT (McCann and Pearlman, 1990). Experiences of emotional transference have also been reported by therapists working with sexually abused children (Possick et al., 2015). However, unlike health professionals, criminal lawyers are not specifically trained to deal with trauma or the difficult interactions that can ensue from working with traumatised populations (see James, 2020); highlighting a need for training and psychoeducation on recognising and responding to vicarious trauma and emotional labour, trauma informed practice, and self-care, to both prepare and support legal practitioners working in the criminal justice system (Katz and Haldar, 2016; Buchanan et al., 2017; Jones, 2019; James, 2020; Birze et al., 2022b). Such approaches have shown some promise for reducing VT symptoms in other professional groups (e.g., healthcare workers; Kim et al., 2022) and therefore, may be suitable for adaptation within a criminal justice context.

Interactions in their role with complainants/victims made prosecutors feel a sense of responsibility to work toward a favourable trial outcome, analogous to therapists describing a “sense of mission” for child clients and their families (Flaskas et al., 2007) and to the “burden of justice” reported by jurors in knowing the consequences following their verdict decision (Bornstein et al., 2005). Crown prosecutors described a burden toward their complainants/victims as they became acutely aware of their hardships and suffering through repeated interactions, challenging the professional boundaries of the legal profession.

The research findings indicate that Crown prosecutors are occupationally expected to interact with PTM, particularly involving the graphic depictions of physical and/or sexual violence. Particularly for those with limited previous interactions with crime and the criminal justice system, this had been a confronting experience when starting out in the role. Research has documented how the rise in digital technology (e.g., audio and video recordings, the internet, and social media) has led to an increase in exposure to such material in the administration of justice and the enduring impact that this has on professionals working in the criminal justice system (Kimpel, 2021; Birze et al., 2022a). Consistent with McCann and Pearlman’s (1990) developmental pathways for VT, participants in the current study described how exposure to graphic PTM resulted in negative changes to their perceptions of reality. Further, in common with research involving therapists working with sexually abused children (Pistorius et al., 2008), counsellors (Bell, 2003) and researchers (Williamson et al., 2020), we found that for some prosecutors, working with PTM also garnered an awareness of their own privilege when compared to the experiences of alleged offenders and complainants.

Particularly difficult for participants were cases that held some form of personal connection or relatability, including offence types reminiscent of prosecutors’ own experiences and cases involving children. PTM involving children has also been identified as particularly distressing for police officers, therapists and other criminal lawyers (Powell et al., 2015; Weir et al., 2021), especially for those who are parents (Burns et al., 2008; Possick et al., 2015; Weir et al., 2021). Unlike studies which have identified resolved trauma as enabling people to bring beneficial responses to their work (Bell, 2003; Pistorius et al., 2008; Pearlman and Caringi, 2009), the majority of Crown prosecutors with relevant histories described becoming triggered by resemblances in files to their own traumas and feeling distressed as a result. This is consistent with Pearlman and Mac Ian (1995), who found that trauma history could present as a vulnerability rather than a strength.

Generally, Crown prosecutors passively accepted the inevitability of trauma and its subsequent impact as being the nature of the role. Nakamura and Orth (2005) distinguished between two types of acceptance reactions where stressors are unchangeable: active acceptance, which involves a problem-solving approach, and resigning acceptance, which involves the abandonment of outward-directed actions as well as a loss of hope. A majority of prosecutors appeared to resort to resigning acceptance in their roles, characterised by an element of helplessness in their ability to assist others, in particular complainants and victims. The desire to become desensitised, that most Crown prosecutors reported striving for as a protective measure, can create difficulties in maintaining necessary empathy for complainants. Russell and Brickell (2015) have referred to empathy as a “double edged sword” for helping professionals: simultaneously an asset and a vulnerability in its fostering of emotional connection with others (McCann and Pearlman, 1990; Figley, 1995; Russell and Brickell, 2015). Reminiscent of the “dance of empathy” outlined by McCann and Colletti (1994), Crown prosecutors expressed difficulties being empathetic while also protecting the self and operating in the professional framework that calls for detachment and unemotionality. This emphasises the need for trauma-informed legal practice (see Katz and Haldar, 2016; James, 2020).

The negative impacts of working with PTM on both individuals and their loved ones have also been documented in relation to police officers (Perez et al., 2010; Civilotti et al., 2021), therapists (Bell, 2003; Pistorius et al., 2008) researchers (Moran and Asquith, 2020) and lawyers (Weir et al., 2021), including sleep disturbances, suspicion, paranoia and hypervigilance in the safety of themselves and others. The impacts on relationships that were reported by Crown prosecutors in this research mirrors the results of research with therapists (Pistorius et al., 2008; Ben-Porat and Itzhaky, 2009) and workers in rape-crisis centres (Clemans, 2004), where participants described being less attentive, emotionally unavailable and avoidant of physical intimacy. Therapists working with sexually abused children in Pistorius et al.’s (2008) study expressed at various points in their career making the choice not to be in a relationship or the avoidance of relationships and sex. Further, participants in Clemans’ (2004) study, who routinely interacted with narratives of sexual violence and traumatised women, more explicitly outlined a diminished interest in sex, the inability to enjoy sex with a partner and feeling uncomfortable about sex. Not only do these studies indicate the personal impact of VT on the individual, they emphasise its ability to permeate into every aspect of the personal lives of professionals.

### 5.1. Implications

This study has identified that Crown prosecutors experience significant negative consequences because of their occupational work with PTM. Participants in the current study reported significant disruptions in their lives that impacted on their wellbeing, including recurring intrusive imagery, inability to sleep and heightened levels of paranoia. It is apparent that significant changes to policies and practices are warranted to address these impacts for two key reasons; first, to protect the wellbeing of Crown prosecutors and second, to preserve the integrity of the criminal justice process.

Crown prosecutors in this study struggled to identify and deal with the consequences of trauma work. This, in part, stemmed from the expectation placed on lawyers to remain detached and unaffected through interactions with PTM, as well as the workplace culture that equated emotions to vulnerability. If displaying emotion is seen as an inherent flaw or failure as a legal professional, this may leave lawyers apprehensive about seeking help. Trauma-specific supervision, peer support networks, and having safe and accessible opportunities to access mental health support have all been identified as ways in which organisations can be more VT informed (Kim et al., 2022). Such approaches have been embraced by other helping professions. For example, practitioners in ‘traditional’ human services professions (e.g., psychologists, nurses, therapists) who work with victims of trauma are provided with regular trauma-informed supervision; and while demonstrating its efficacy has proven difficult (Wheeler, 2004), it appears promising in its provision and endorsement of a safe space for reflection, discussion and resolution of workplace concerns, exploration of alternative perspectives (Berger and Quiros, 2016; Zwisohn et al., 2018), and recognition and normalisation of professionals’ emotional reactions to PTM (Wilson et al., 2016). Access to confidential, externally provided mental health support, not only when experiencing VT or negative psychological symptoms, but for ongoing self-care may also be beneficial and promote an open workplace culture that is VT informed (Zwisohn et al., 2018).

A Crown prosecutor labouring under the strains of VT may inadvertently cause harm to those engaged in the criminal justice process. As discussed by the participants, real and significant consequences exist at the heart of the criminal justice system, such as the loss of liberty through the imposition of stringent bail conditions or imprisonment for defendants, or the lost opportunity for redress for complainants. This can lead to the re-traumatisation of already vulnerable individuals if Crown prosecutors do not have the capacity to follow due process or set professional boundaries. Thus, it is imperative that Crown prosecutors are provided with access to appropriate tools and support services and encouraged to prioritise and maintain their wellbeing so they are able to effectively carry out their duties. The provision of education, training and support that inherently recognises the existence of VT could assist in fostering safe environments and empower Crown prosecutors to identify, disclose and address the adverse consequences of working with PTM.

### 5.2. Strengths and limitations

This is the first study in New Zealand to qualitatively explore the experiences of Crown prosecutors in working with PTM. Through conducting qualitative research in a field where emphasis has been placed on quantitative explorations, rich and detailed data was obtained that reflected the subjective experiences of the prosecutors, in their own words. Data was collected from different firms across New Zealand in terms of size, locations (e.g., urban, provincial) and from Crown prosecutors of different experience levels, gender identities and ethnicities to ensure a range of views, experiences and perspectives were gathered and to ensure information power. Although effort was made in the recruitment process to hear from a diverse range of voices, in terms of gender, ethnicity and experience level from various sized firms, this was difficult to achieve. Given the time constraints, not all Crown Solicitor firms were able to be approached for recruitment.

Further, while having some commonalities, each jurisdiction operates under different criminal justice structures and systems. Thus, the current findings for Crown prosecutors only reflect the experiences of those working in the New Zealand context, due to differences in workplace factors such as scope of duties, staffing and culture. However, several lawyer-specific vulnerabilities have been identified cross-culturally – e.g., high stances of drug and alcohol dependency (Krill et al., 2016) and compromised mental wellbeing (e.g., Leclerc et al., 2020; Weir et al., 2021). While prosecutors in other jurisdictions may not undertake exactly the same role as New Zealand Crown prosecutors, they all work within criminal justice settings that expose them to PTM as well as distressed and vulnerable populations. Accordingly, there may be some common experiences that can be extrapolated from the experiences of Crown prosecutors reported in this study.

### 5.3. Directions for future research

There is some evidence to suggest the impact of occupational factors other than exposure to PTM in the development of VT, such as the number of trauma cases professionals are assigned (van Minnen and Keijsers, 2000; Devilly et al., 2009). For example, Devilly et al. (2009) in their study of mental health professionals found exposure to patients’ traumatic material did not affect STS or VT. Rather, work-related stressors were found to best predict participant distress. Although these findings must be viewed in the context of the distinct workplace framework of mental health professionals (who generally have better training and support around trauma) compared to legal professionals, it is still important to further explore workplace factors for Crown prosecutors to determine the extent of their influence on wellbeing and possible etiological pathways for VT. Whilst occupational exposure to PTM is inevitable in the role of Crown prosecutors, workplace factors are more amenable to change (e.g., better workload allocation, provision of support services) and can alleviate the stress of working with PTM. Thus, there is merit in exploring the impact of these workplace factors in further detail.

Further, the current research has predominantly focused on the negative aspects of working with trauma. However, there is growing scholarship on the positive changes following trauma work, focusing on vicarious post-traumatic growth following occupational exposure to PTM (e.g., Barrington and Shakespeare-Finch, 2013; Cohen and Collens, 2013; Ben-Porat, 2015). In pursuing this line of research, researchers must be wary that the existence of VT and vicarious post-traumatic growth are not mutually exclusive (Bober and Regehr, 2006). Instead, the pathway of negative or positive impacts are determined by consistent personal and supervisory management of VT (Barrington and Shakespeare-Finch, 2013; Pack, 2014). Implementation and evaluation of trauma-informed practices would normalise reactions and enable lawyers to make informed decisions in their work and self-care, could assist in the creation of a safe and supportive work environment, and permit Crown prosecutors to “survive and thrive” in their roles (James, 2020).

## 6. Conclusion

In exploring the lived experiences of nineteen New Zealand Crown prosecutors, this study demonstrated that Crown prosecutors occupationally experience VT through working with PTM in their roles. The presentation of VT among the participants was largely negative and ranged in its persistence and severity. Participants described the subsequent impacts on both their personal and professional lives. Most significantly, the findings indicate that participants lacked the confidence and support structures in the workplace to identify and appropriately combat the impacts of VT.

This study adds to the limited qualitative research conducted among professionals working in the criminal justice system and aims to provide a deeper and richer understanding of how VT can impact professionals persistently engaged with trauma. The hope is for further research to be conducted to incorporate a number of different levels of policy and practice to proactively *“help the helpers”* (Helm, 2014). In the criminal context, where emotion is rarely “so easily quarantined”, the impact of trauma cannot be overlooked (Baillot et al., 2013). Crown prosecutors are an integral mechanism of the criminal justice system and there is an overarching duty of care to ensure they are not carrying out their work to their own detriment.

## Data availability statement

The datasets presented in this article are not readily available because the confidential nature of the data. Requests to access the datasets should be directed to NT, nichola.tyler@vuw.ac.nz.

## Ethics statement

The studies involving human participants were reviewed and approved by Victoria University of Wellington Human Ethics Committee. The patients/participants provided their written informed consent to participate in this study.

## Author contributions

RK: conceptualization, methodology, formal analysis, writing—original draft. NT: conceptualization, methodology, formal analysis, writing—review and editing, supervision. YT: conceptualization, methodology, formal analysis, writing—review and editing, supervision. All authors contributed to the article and approved the submitted version.
