# Corrigendum: “Wading through the worst that humanity does to each other”: New Zealand Crown prosecutors' experiences of working with potentially traumatic material in the criminal justice system

**DOI:** 10.3389/fpsyg.2024.1496938

**Published:** 2024-10-15

**Authors:** Rachel Kim, Nichola Tyler, Yvette Tinsley

**Affiliations:** ^1^School of Psychology, Victoria University of Wellington, Wellington, New Zealand; ^2^Faculty of Law, Victoria University of Wellington, Wellington, New Zealand

**Keywords:** vicarious trauma, potentially traumatic material, Crown prosecutors, legal professionals, lawyers, qualitative

In the published article, there was an error in Table 1. The labelling of the rows for gender were inverted. Table 1 should read the same as the corresponding text under Section 3.2 participants - 7 male (36.8%) and 12 (63.2%) female participants. The corrected Table 1 and its caption *Participant demographics* appear below.

**Table 1:** Participant demographics.

**Demographics**	**Category**	***N***	**%**
Gender	Male	7	36.8
	Female	12	63.2
Ethnicity	Pākehā/European	16	84.2
	Māori	2	10.5
	Asian	1	5.3
Classification^1^	Junior	9	47.4
	Intermediate	4	21.1
	Senior	6	31.6
Provincial/urban	Provincial	12	63.2
	Urban	7	36.8

^1^The full classification criteria for a junior, intermediate and principal prosecutor can be found in the Crown Solicitors: Terms of Office (2017).

The authors apologize for this error and state that this does not change the scientific conclusions of the article in any way. The original article has been updated.

